# P-1401. Association Between Intimate Partner Violence and Antiretroviral Adherence Among Pregnant Women Living with HIV

**DOI:** 10.1093/ofid/ofae631.1576

**Published:** 2025-01-29

**Authors:** Aasith Villavicencio Paz, John B Jemmott, Fatemeh Ghadimi, Hervette Nkwihoreze, Sara Seyedroudbari, William R Short, Aadia Rana, Anandi N Sheth, Rachel Scott, Gweneth Lazenby, Rodney Wright, Florence Momplaisir

**Affiliations:** Hospital of the University of Pennsylvania, Philaldelphia, Pennsylvania; Department of Psychiatry and Annenberg School for Communication, University of Pennsylvania, Philadelphia, Pennsylvania; University of Pennsylvania, Bronx, New York; University of Pennsylvania, Bronx, New York; University of Pennsylvania, Bronx, New York; University of Pennsylvania, Bronx, New York; University of Alabama-Birmingham Heersink School of Medicine, Birmingham, AL; Emory University School of Medicine, Atlanta, Georgia; MedStar Health Research Institute, Washington, DC; MUSC, Charleston, South Carolina; Montefiore, Bronx, New York; University of Pennsylvania, Bronx, New York

## Abstract

**Background:**

Despite increased access to antiretroviral therapy (ART) for women with HIV (WWH), challenges like unaddressed mental health issues and poor postpartum HIV care retention persist. This analysis evaluates whether Intimate Partner Violence (IPV) is related to reduced ART use and adherence, a topic not extensively studied in the US, especially in pregnant WWH.

**Figure:**
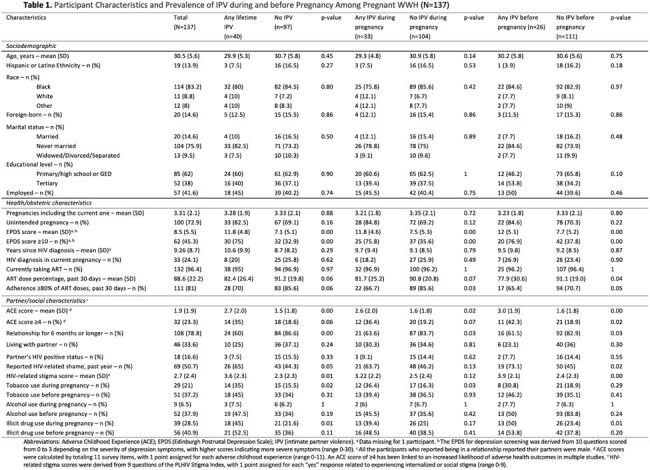

Participant Characteristics and Prevalence of IPV during and before Pregnancy Among Pregnant WWH (N=137)

**Methods:**

We analyzed secondary data from a multisite US randomized trial of a peer-led behavioral intervention to improve postpartum retention in WWH. Data was collected from the baseline survey: socio-demographic, health, and psychosocial characteristics, using tools such as the Edinburgh Postnatal Depression Scale (EPDS), Adverse Childhood Experiences (ACE) and HIV-related stigma scores, and the WHO Violence Against Women questionnaire to assess IPV. A multivariable logistic regression (MLR) examined associations between IPV timing (lifetime, before or in pregnancy) and type (physical, emotional, or sexual) and ART adherence (self-reported, ≥ 80% of prescribed ART doses in the prior month), adjusting for potential covariates.

**Figure:**
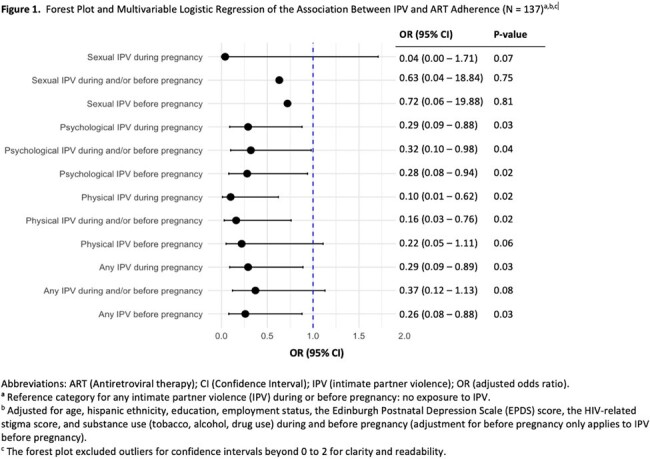

Forest Plot and Multivariable Logistic Regression of the Association Between IPV and ART Adherence (N = 137)

**Results:**

A total of 137 pregnant WWH enrolled between March 2020 and March 2024 were included: mean age was 30.5 (SD 5.6); 83% were Black, 14% Hispanic; mean number of pregnancies was 3.31 (SD 2.1); mean number of years with HIV 9.26 (SD 8.7). Twenty women reported not disclosing their HIV status to their partners, 14 due to fear of abandonment, and two due to fear of physical violence. Depression, stigma, and ACEs were prevalent: an EPDS score of ≥ 10 was seen in 45% of women, an ACE score of ≥ 4 in 23%, and 51% reported HIV-related shame. Forty women (29%) reported any lifetime IPV exposure (39, psychological; 13, physical; 4, sexual). Significantly higher EPDS, ACE, and stigma scores were seen in women exposed to IPV (p < 0.02). Physical IPV during pregnancy had the strongest association with decreased ART adherence in pregnancy (aOR=0.10, p=0.02). Psychological IPV and any IPV type during or before pregnancy were also associated with lower odds of adherence in pregnancy.

**Conclusion:**

We found high IPV rates and a significant negative association with ART adherence among pregnant WWH. These findings highlight the importance to screen for and address IPV in HIV care to improve maternal and child health outcomes.

**Disclosures:**

**William R. Short, MD**, Gilead: Grant/Research Support|Janssen: Honoraria|ViiV Healthcare: Advisor/Consultant|ViiV Healthcare: Honoraria **Rachel Scott, MD,MPH,FACOG**, DHHS Perinatal Guidelines: Board Member|UW STD Prevention Training Center (UW STD PTC): Honoraria|ViiV Healthcare: Advisor/Consultant|ViiV Healthcare: Grant/Research Support|Vindico CME: Honoraria

